# Maternal-related deaths and impoverishment among adolescent girls in India and Niger: findings from a modelling study

**DOI:** 10.1136/bmjopen-2016-011586

**Published:** 2016-09-23

**Authors:** Stéphane Verguet, Arindam Nandi, Véronique Filippi, Donald A P Bundy

**Affiliations:** 1 Department of Global Health and Population, Harvard T.H. Chan School of Public Health, Boston, Massachusetts, USA; 2 Center for Disease Dynamics, Economics and Policy, Washington, DC, USA; 3 Tata Centre for Development, Harris School of Public Policy, University of Chicago, Chicago, Illinois, USA; 4 Department of Infectious Disease Epidemiology, London School of Hygiene and Tropical Medicine, London, UK; 5 Bill & Melinda Gates Foundation, Seattle, Washington, USA

**Keywords:** PUBLIC HEALTH, REPRODUCTIVE MEDICINE, HEALTH ECONOMICS

## Abstract

**Background:**

High levels of maternal mortality and large associated inequalities exist in low-income and middle-income countries. Adolescent pregnancies remain common, and pregnant adolescent women face elevated risks of maternal mortality and poverty. We examined the distribution across socioeconomic groups of maternal deaths and impoverishment among adolescent girls (15–19 years old) in Niger, which has the highest total fertility rate globally, and India, which has the largest number of maternal deaths.

**Methods:**

In Niger and India, among adolescent girls, we estimated the distribution per income quintile of: the number of maternal deaths; and the impoverishment, measured by calculating the number of cases of catastrophic health expenditure incurred, caused by complicated pregnancies. We also examined the potential impact on maternal deaths and poverty of increasing adolescent girls' level of education by 1 year. We used epidemiological and cost inputs sourced from surveys and the literature.

**Results:**

The number of maternal deaths would be larger among the poorer adolescents than among the richer adolescents in Niger and India. Impoverishment would largely incur among the richer adolescents in Niger and among the poorer adolescents in India. Increasing educational attainment of adolescent girls might avert both a large number of maternal deaths and a significant number of cases of catastrophic health expenditure in the 2 countries.

**Conclusions:**

Adolescent pregnancies can lead to large equity gaps and substantial impoverishment in low-income and middle-income countries. Increasing female education can reduce such inequalities and provide financial risk protection and poverty alleviation to adolescent girls.

**Strengths and limitations of this study**

- One strength of the study is the detailed examination of the distribution of maternal deaths and impoverishment among pregnant adolescent girls per socioeconomic group.
- Another strength is the use of the extended cost-effectiveness analysis methodology to point to the large equity and poverty gaps related to adolescent pregnancies.
- One limitation of the study is the use of secondary epidemiological and cost data extracted from surveys and the literature, and the lack of observational data.

## Introduction

In spite of substantial progress with respect to the United Nations Millennium Development Goal 5A—to reduce the maternal mortality ratio (number of maternal deaths per 100 000 live births) by three-quarters between 1990 and 2015—there still exist wide disparities in maternal mortality worldwide.^1–3^ Maternal mortality ratios remain high in sub-Saharan Africa, notably in West Africa, and large numbers of maternal deaths still occur in Southern Asia in densely populated countries such as India.^2^ ^3^ Together, sub-Saharan Africa and Southern Asia, about 38% of the world's population,^4^ accounted for 88% of the world's maternal deaths in 2015.^3^

Young adolescent women face high risks of maternal mortality in low-income and middle-income countries.^5–7^ Adolescent girls under age 16 can have a risk of maternal mortality up to five times higher as compared with women aged 20–24 years.^7^ Though expansion of female education and labour participation (ie, economic opportunity) have occurred worldwide,^8^ early marriages are still common in many countries, with up to 50% and 80% of women being married by age 18 in India and Niger, respectively, for example.^9^ As a result, the rates of adolescent pregnancies remain high in many low-income and middle-income countries.^10^ ^11^

Maternal and adolescent health must also be viewed through a wider lens beyond mortality, notably considering morbidity outcomes such as long-term sequelae for mothers and their children, and crucially the financial vulnerability of women and adolescent girls.^12^ ^13^ For instance, pregnant young adolescents present higher chances of school dropouts^14^ and may face high risks of impoverishment and negative economic consequences^15^ ^16^ when they carry their pregnancy to term. Indeed, out-of-pocket (OOP) payments for medical care can lead to impoverishment in many low-income and middle-income countries, and healthcare expenditure can often be ‘catastrophic’—defined as exceeding a certain fraction of total consumption expenditure.^17^

Protection from healthcare financial risks has become a critical component of national strategies in many countries.^18^ ^19^ The reduction of these financial risks is one objective of public sector policies. For example, by increasing education levels of young girls, pregnancies could be reduced and their associated risks of mortality and impoverishment, especially among the poorest, could be decreased.

Health economic evaluations have mostly focused on estimating the cost of an intervention per health gain (eg, cost per disability-adjusted life year averted).^20^ Extended cost-effectiveness analysis (ECEA)^21–23^ supplements such traditional economic evaluations with equity (eg, distribution of health outcomes per socioeconomic group) and financial risk protection (prevention of medical impoverishment) evaluation. In this respect, it can address questions related to policies to be implemented for increasing financial risk protection, promoting poverty alleviation and equity, and improving the distribution of health in countries.

In this paper, consistent with ECEA, we first quantified the maternal-related deaths and medical impoverishment consequences of adolescent pregnancies in two countries: Niger, with the highest total fertility rate worldwide; and India, with the largest number of maternal deaths globally. We then estimated the potential reduction in adolescent maternal-related deaths, and equity and financial risk protection benefits that could be gained through increased educational attainment of young girls in these two countries.

## Methods

### Modelling approach of the study

We considered the population of adolescent women, aged 15–19 years, in two countries, namely, Niger and India. Niger has the highest total fertility rate globally (7.6) and a high maternal mortality ratio (553 per 100 000 live births) leading to 5400 maternal deaths annually. India presents the largest population in Southern Asia (1.3 billion); it has the largest number of maternal deaths worldwide (45 000 deaths) and a high maternal mortality ratio (174 per 100 000 live births).^2–4^

#### Adolescent maternal-related deaths, OOP costs and impoverishment

We quantified per income quintile and per year: (1) the number of adolescent maternal deaths; (2) the total OOP costs induced by complicated maternal deliveries; and (3) the impoverishment caused by complicated deliveries, using an estimated number of cases of catastrophic health expenditure among adolescent women. Complicated deliveries included assisted vaginal deliveries with obstetric complications (eg, haemorrhage, eclampsia) and caesarean sections.

First, we estimated the number of maternal deaths related to adolescent pregnancies. It was based on the maternal mortality ratio (174 and 553 per 100 000 live births among 15–49 years old women in India and Niger, respectively), which was distributed across the five adolescent ages (15, 16, 17, 18, 19), based on the relative risk (compared with 20–24 years old women) of maternal mortality among adolescents (4.6, 1.0, 1.0, 1.0, 1.0; table 1) and the per cent of women aged 15, 16, 17, 18 and 19 pregnant (1%, 3%, 5%, 9%, 12% in India and 3%, 12%, 16%, 19%, 18% in Niger; table 1). We then assigned these adolescent deaths to five income quintiles using the distribution of adolescent pregnancies per income quintile (19%, 17%, 13%, 8%, 3% in India and 41%, 43%, 37%, 32%, 19% in Niger; table 1). Further detail is given in the online supplementary appendix section 1.1.

**Table 1:** Parameters used for the analysis of adolescent maternal deaths and impoverishment in India and Niger

Parameter	India	Niger	Sources
Total population	1311 millions	20 millions	4
Population of women aged 15–19	58 400 000	1 021 000	4
Maternal mortality ratio (per 100 000 live births among 15–49 years old)	174	553	3
Occurrence of complicated maternal delivery (among all deliveries)	15%	15%	Authors' assumption^42^
Relative risk of maternal mortality for women aged 15, 16, 17, 18 and 19	4.6, 1.0, 1.0, 1.0, 1.0	4.6, 1.0, 1.0, 1.0, 1.0	Based on^7^
Per cent of women aged 15–19 pregnant, from poorest to richest (income quintile 1–5)	19%; 17; 13; 8; 3	41%; 43; 37; 32; 19	26 27
Per cent of women aged 15, 16, 17, 18 and 19 pregnant	1%; 3; 5; 9; 12	3%; 12; 16; 19; 18	26 27
Healthcare usage (skilled birth attendance coverage), from poorest to richest (income quintile 1–5)	24%; 34; 48; 64; 85	13%; 19; 22; 30; 71	26 28
Out-of-pocket direct medical cost of complicated delivery, from poorest to richest (income quintile 1–5)	$58; $62; $70; $81; $108	$97; $127; $140; $124; $152	Based on^15^ ^29^ ^31^
Out-of-pocket transportation cost, from poorest to richest (income quintile 1–5)	$8; $8; $8; $8; $6	$4	Based on^30^ ^31^
Gross domestic product per capita	$1596	$427	10
Gini index	0.34	0.32	10
Impact of female education on adolescent pregnancy rate	1 additional year of education leads to an 18% relative reduction (SE=2%) in the adolescent pregnancy rate	1 additional year of education leads to an 18% relative reduction (SE=2%) in the adolescent pregnancy rate	Online supplementary appendix section 2 and table S2
Cost of primary education per pupil per year	$258	$72	Based on^33^

All costs are expressed in 2014 US$.

10.1136/bmjopen-2016-011586.supp1 Supplementary appendix

Second, we estimated the amount of OOP costs related to complicated deliveries and associated transportation costs per income quintile. It was based on the occurrence of complicated deliveries (15%; table 1), the relative risk of maternal mortality among adolescents, and the per cent of women aged 15, 16, 17, 18 and 19 pregnant. We then assigned these OOP costs to five income quintiles using both the distribution of adolescent pregnancies per income quintile and the distribution of healthcare usage per quintile (19%, 29%, 42%, 58%, 80% in India and 13%, 19%, 22%, 30%, 71% in Niger; table 1). Further detail is given in the online supplementary appendix section 1.2.

Third, medical impoverishment was quantified by the estimated number of cases of catastrophic health expenditure incurred, which depended on assumed individual income and OOP costs. A case of catastrophic expenditure was counted when OOP costs were found to be higher than 10% of individual income, a commonly used threshold in the literature.^17^ Hence, we derived a distribution of income drawn from a simulated gamma distribution whose shape and scale parameters were based on gross domestic product per capita ($1596 for India and $427 for Niger, in 2014; table 1) and Gini coefficient (0.34 and 0.32, respectively; table 1).^10^ ^24^ ^25^ For each complicated delivery incurring OOP costs, we assigned an annual income extracted from the income distribution. The annual income was also used to define the income quintile to which each individual belonged (table 1). We estimated the number of adolescents, per income quintile and per age (15, 16, 17, 18, 19), for whom the size of OOP costs (sum of direct medical costs and transportation costs) would exceed 10% of their income. Further detail is given in the online supplementary appendix section 1.3.

#### Data sources

We relied on secondary data extracted from estimates from international agencies, surveys and the published literature. We used country maternal mortality and population estimates from the United Nations for the year 2015.^2–4^ The percentage of women aged 15–19 who were pregnant per income quintile and skilled birth attendance coverage (proxy for healthcare usage) per income quintile were obtained from Niger's 2012 Demographic and Health Survey and India's 2007–2008 District Level Household Survey and 2005–2006 National Family Health Survey.^26–28^ We used an estimated relative risk (compared with 20–24 years old women) of maternal mortality among adolescents (15, 16, 17, 18, 19 years old)^7^ and data on OOP costs for complicated deliveries and transportation costs which were extracted from the literature for Niger^15^ ^29^ ^30^ (dating from 2006, and from 2008 to 2011), and from India's 2004 National Sample Survey^31^ (see table 1 for all the inputs and online supplementary appendix section 1 for details on the estimation).

### Impact of education on adolescent maternal-related deaths and impoverishment

We simulated the hypothetical impact of a 1-year increase in the education level of young girls. Assuming an association with the adolescent pregnancy rate, we estimated the potential reduction in adolescent maternal-related deaths and impoverishment. We calculated the number of maternal deaths averted due to a decrease in adolescent pregnancies, the amount of OOP costs averted due to the prevented complicated deliveries, and subsequently the number of cases of catastrophic health expenditure averted. The counterfactual scenario corresponded to the case where female education was maintained at the same level; hence, there would be no change in the adolescent pregnancy rate.

We studied the linear relationship between the mean number of years of education among women aged 15–44^32^ and the adolescent pregnancy rate (percentage of women aged 15–19 who have had children or are currently pregnant) among low-income and middle-income countries with a population greater than one million.^4^ This allowed us to estimate what might be the reduction in adolescent pregnancies with expansion of female education (table 1). Further detail is given in the online supplementary appendix section 2. Subsequently, we could quantify per income quintile: the number of maternal deaths averted; the OOP costs averted among complicated deliveries averted and the number of cases of catastrophic expenditure averted.

We also tentatively assessed the costs associated with raising by 1 year the education level of young girls. To do so, we multiplied the entering female adolescent cohort (estimated as the population of women aged 15–19 divided by five, or about 204 000 in Niger for example) by the annual cost of primary education per pupil as estimated by the United Nations Educational, Scientific and Cultural Organization for 2012.^33^ This enabled us to quantify what might be the financial resources needed to achieve such an increase in female education. We did not discount the costs and benefits as the events would occur only a few years into the future. Yet we conducted a sensitivity analysis (see online supplementary appendix tables S8 and S9) where benefits and costs were discounted at 3% per year over 5 subsequent years (corresponding to the ages 15, 16, 17, 18 and 19).

All costs were expressed in US$2014 using the World Bank's time series for official exchange rates and US consumer price index.^10^ Complete details on the mathematical derivations used are given in the online supplementary appendix sections 1–3, and we used R statistical software (http://www.r-project.org) for all analyses.

### Sensitivity analysis

We assessed the robustness of our findings using both univariate and probabilistic sensitivity analysis.

First, a probabilistic sensitivity analysis was conducted using Monte Carlo simulations (n=1000 trials) where all key parameters (maternal mortality ratio, relative risk of mortality, costs, impact of mean years of female education on adolescent pregnancy rate) were varied simultaneously. Parameter uncertainty was included through sampling n values for each parameter to which was assigned a gamma, beta or logistic distribution built on each input's mean and standard deviation resulting in n samples. Extracting the 2.5 and 97.5 percentiles allowed the determination of 95% uncertainty ranges (URs), which are reported with the results. Further details are given in the online supplementary appendix section 4.

Second, three univariate sensitivity analyses were performed including (1) different thresholds (20% and 40% of income) for the catastrophic expenditure; (2) a poverty headcount (estimating the number of individuals falling below the country poverty line due to OOP costs) in lieu of cases of catastrophic expenditure; and (3) a smaller effect, 11% relative reduction (instead of 18%; see online supplementary appendix table S2), for the impact of a 1-year increase in female education on adolescent pregnancy rate.

## Results

**Figure 1:**
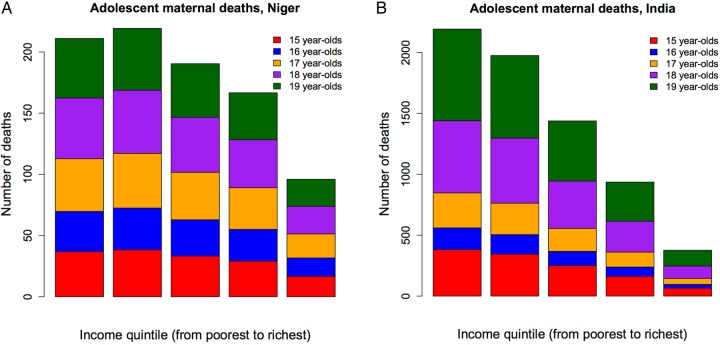
Estimated number of maternal deaths among 15–19 years old women in Niger (A) and India (B), per income quintile.

The estimated annual number of adolescent maternal deaths was 6920 (95% UR 5110–8870) in India and 880 (590–1250) in Niger (figure 1). The population of adolescent women in the two countries, about 58 million in India and around 1 million in Niger, was responsible for the large difference in magnitude between the two countries. We observed that there was a significant variation across income quintiles. In India, the bottom two income quintiles would incur about 60% (95% UR 47–80%) of the adolescent maternal deaths, and in Niger the bottom two quintiles would incur about 49% (34–73%) of the deaths. In both countries, the deaths would largely accrue among the poor, which points to the need for tailored policies that target disadvantaged and marginalised populations.

**Figure 2:**
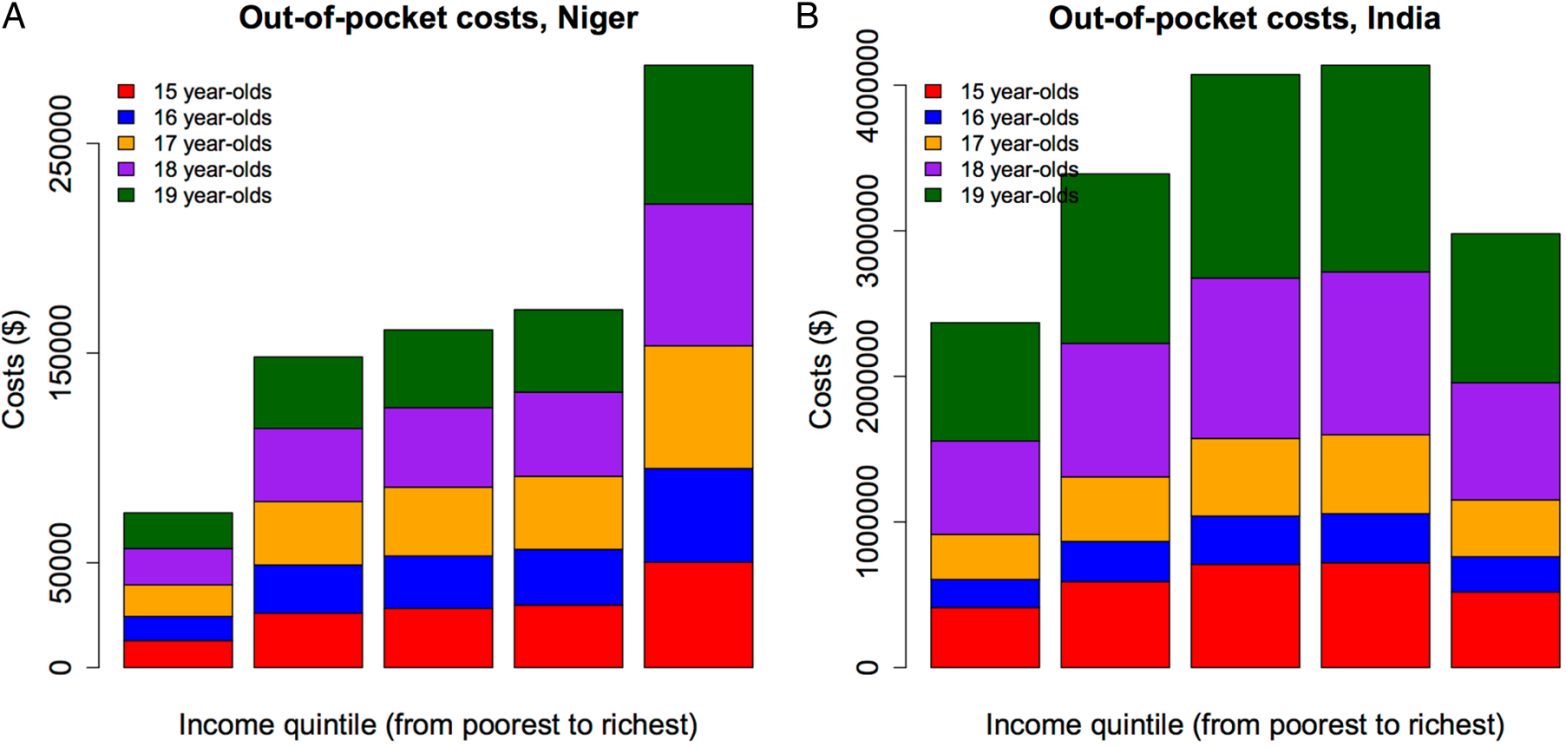
Estimated out-of-pocket costs among 15–19 years old women in Niger (A) and India (B), per income quintile.

The annual OOP costs were estimated at $17 million (95% UR 14–20) in India and $840 000 ($690 000–$1 000 000) in Niger (figure 2). In India, the bottom two income quintiles would capture about 34% (95% UR 28–41%) of all OOP costs, while in Niger the bottom two quintiles would represent around 26% (22–32%) of all costs. Contrary to the deaths, the OOP costs would accrue slightly more among the richer quintiles in both countries, mimicking the higher healthcare usage among richer quintiles (table 1). The poor use health services less notably because of financial constraints (eg, risk of large OOP costs, cannot afford to leave work). Therefore, adapted public policies (eg, cash transfers) should be designed to address such economic barriers.

**Figure 3:**
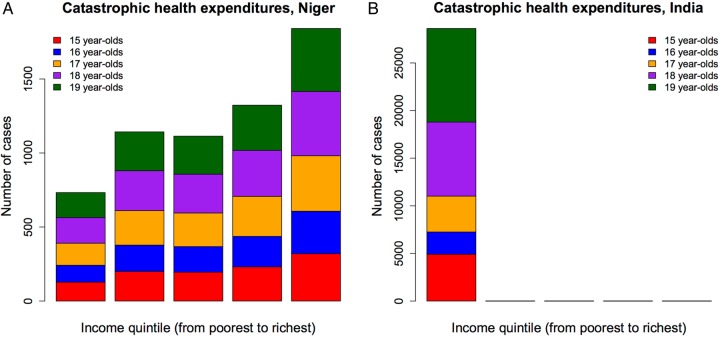
Estimated cases of catastrophic health expenditures among 15–19 years old women in Niger (A) and India (B), per income quintile.

The annual number of cases of catastrophic health expenditure was estimated at 28 620 (95% UR 16 180–52 590) in India and 6150 (5370–6900) in Niger (figure 3). Overall, this represented about a 0.4% (95% UR 0.2–0.7%) incidence among Indian pregnant adolescent girls and around a 1.8% (1.5–2.0%) incidence among pregnant adolescent girls in Niger. In India, the bottom quintile captured all of the cases of catastrophic expenditure, while in Niger the bottom two quintiles represented about 30% (95% UR 27–35%) of all cases. Per capita of pregnant adolescent, Niger exhibited a fivefold higher incidence in catastrophic expenditure. Catastrophic expenditures were accruing across all quintiles in Niger, but only among the poorest quintile in India. Such differences can be explained somewhat by India being much richer than Niger with a four times higher national income (table 1). These findings express the necessity to ensure free maternal care for all in Niger and for the poorest in India.

**Figure 4:**
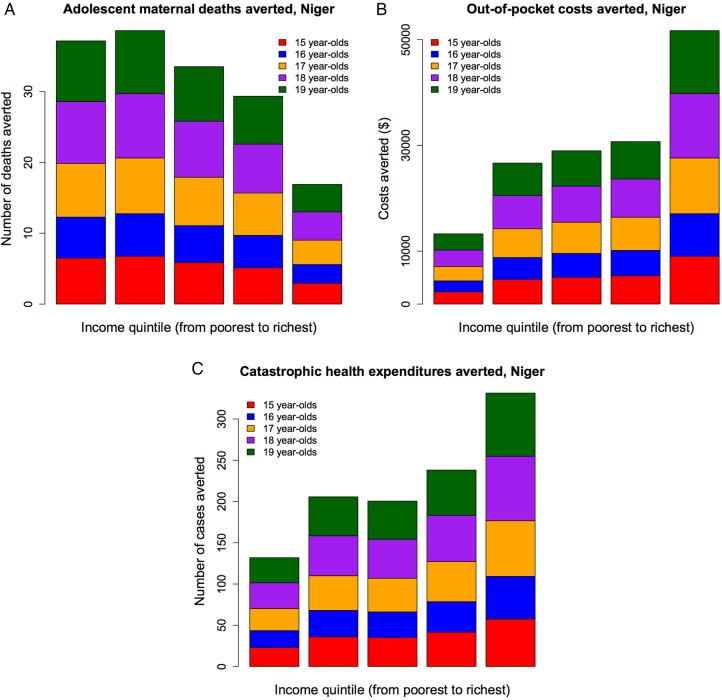
Impact of increasing mean years of female education by 1 year in Niger: number of adolescent maternal deaths averted (A), amount of adolescent out-of-pocket costs averted (B) and number of adolescent cases of catastrophic health expenditures averted (C), per income quintile.

**Figure 5:**
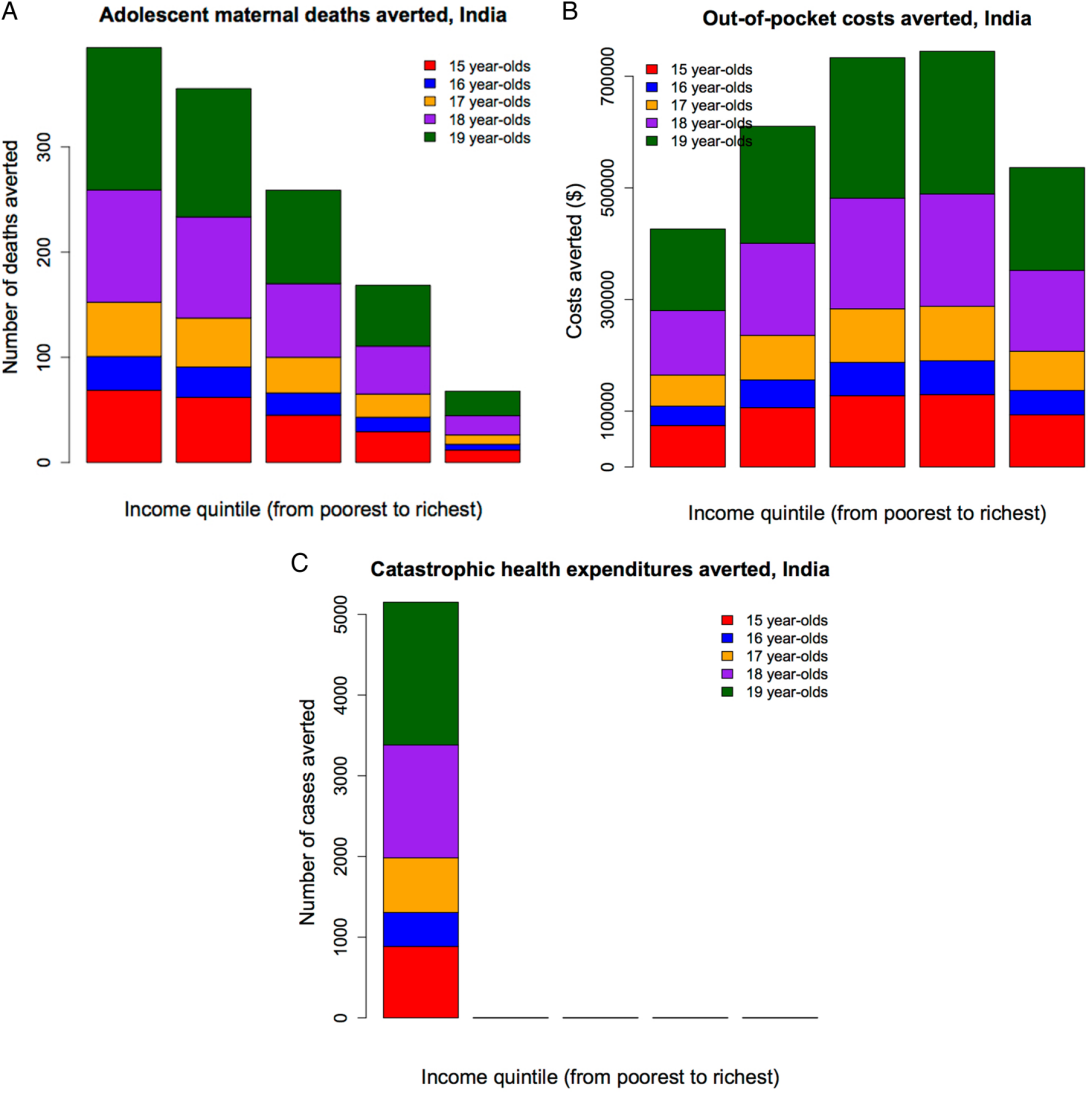
Impact of increasing mean years of female education by 1 year in India: number of adolescent maternal deaths averted (A), amount of adolescent out-of-pocket costs averted (B) and number of adolescent cases of catastrophic health expenditures averted (C), per income quintile.

By increasing female mean years of education by 1 year, the adolescent maternal-related deaths averted would be 1200 (95% UR 800–1700) in India and 160 (100–240) in Niger; the OOP costs averted would be $3 050 000 (95% UR $2 270 000–$3 900 000) in India and $150 000 ($110 000–$200 000) in Niger; and the cases of catastrophic expenditure averted would be 5150 (95% UR 2730–9200) in India and 1110 (840–1430) in Niger (figures 4 and 5). In each country, the extent of maternal deaths averted, OOP costs averted and catastrophic expenditure averted varied greatly across quintiles. In both countries, more adolescent lives would be saved among the bottom two quintiles (out of all lives saved: 60% (95% UR 44–88%) in India and 49% (32–78%) in Niger), compared with the top two quintiles (out of all lives saved: 19% (14–28%) and 30% (20–48%), respectively). In contrast, in Niger, there were more OOP costs averted among the richer, with about 54% (95% UR 41–73%) of total OOP costs averted among the top two quintiles as opposed to only 26% (20–35%) among the bottom two quintiles. This was largely due to the fact that richer individuals used more healthcare than poorer individuals. In India, the OOP costs averted were more evenly distributed: about 42% (95% UR 32–57%) of total OOP costs averted accrued among the top two quintiles as opposed to 34% (26–46%) among the bottom two quintiles. As regards financial risk protection (cases of catastrophic expenditure averted), the results reflected a combination of key drivers including the distribution of healthcare usage and individual income. For example, Niger saw a larger number of cases of catastrophic expenditure averted among the richer (51%, 95% UR 41–67%, of all cases averted accrued among the top two quintiles) than the poorer (30%, 95% UR 24–40%, of all cases averted accrued among the bottom two quintiles), as there were large inequalities among healthcare usage (13% among the poorest, 71% among the richest) and as Nigeriens' income was very low even among the richer socioeconomic groups (Niger's national income is $427 only). In contrast, India saw all cases of catastrophic expenditure averted among the poor (100% within the bottom quintile), as in spite of large inequalities in usage (24% among the poorest, 85% among the richest), there were still substantial income disparities in the country (with a national income of $1596), and therefore richer individuals would face little risk of catastrophic expenditure (figures 4 and 5). As a result of increasing female education uniformly across socioeconomic groups, about a fifth of adolescent maternal-related deaths could be averted, and 50–60% of those would accrue among the poor in both countries. Hence, universal girl education is largely pro-poor in terms of health benefits. About a fifth of cases of catastrophic expenditure would be prevented. All those cases prevented would accrue to the poorest in India, which reinforces the pro-poor aspect of universal girl education; whereas a majority of them would accrue to the richer income groups in Niger, which points to both the large inequalities in access to care and the poor disposable incomes across the whole Nigerien population.

The total costs of increasing female mean years of education by 1 year were estimated at $15 million (95% UR 10–20) in Niger and $3011 million (2109–4055) in India. When the threshold used for the estimation of cases of catastrophic health expenditure was raised (to 20% or 40%), expectedly, the magnitude of the cases incurred decreased in India and Niger, with a slight alteration of the distribution across quintiles in Niger. Alternatively, when the poverty headcount metric was used, the distribution of induced poverty across quintiles was significantly altered (see online supplementary appendix tables S4 and S5). Finally, when the impact of female education on adolescent pregnancy rate was reduced (to 11% in lieu of 18%), the maternal deaths, OOP costs and impoverishment averted were all reduced by 39% (see online supplementary appendix tables S6 and S7).

## Discussion

We quantified the maternal deaths and impoverishment incurred among adolescent girls in Niger and India, as well as the impact of increasing girl education on reducing such maternal deaths and impoverishment. Our approach examines the distributional aspects per socioeconomic group of health outcomes and impoverishment and provides critical metrics of equity and poverty for policymakers when allocating financial resources. As a case in point, we showed that, by avoiding early pregnancies, increasing girl education could bring poverty alleviation benefits, which points to the great economic vulnerability of adolescent women in such settings.^12^ ^13^

Beyond the large mortality and financial outcomes, there are wide variations across socioeconomic groups. On the one hand, more maternal deaths would occur among the poorer groups as they face higher rates of early pregnancy. On the other hand, more OOP costs would be incurred among the richer groups as richer individuals use more healthcare than poorer individuals. Indeed, usage increases with income, from 19% (13%) in the bottom income quintile to 80% (71%) in the top quintile in India (Niger); OOP direct medical costs increase slightly with income, from $58 ($97) in the bottom quintile to $108 ($152) in the top quintile in India (Niger; table 1). Notably, the large inequalities in usage can be due to a variety of factors including financial (eg, expensive care and unaffordability to leave work for individuals and caregivers) and social (eg, cultural acceptability in rural areas) factors. Individual income and country wealth, as well as low income versus middle income, also affected the distribution of the poverty outcomes.

Nevertheless, our analysis presented several limitations. Most importantly, we had limited data and relied on secondary data and the published literature. Indeed, we inferred a relationship between female years of education and adolescent pregnancy rates. Yet there might be issues including missing evidence that this relationship only occurred in the direction assumed, in spite of several studies (in the focus countries of this study and elsewhere) having established links between education and fertility either directly or through intermediate outcomes.^34–37^ Likewise, a more comprehensive accounting of incurred costs for the adolescent women could be included, with detailed accounting of medical costs, transportation and housing costs, and time and wages lost. For simplicity, we used average OOP expenses linked to complicated deliveries, whereas OOP costs might significantly rise with the degree of complication and emergency (eg, caesarean section), for example. In particular, there are limitations associated with India's National Sample Survey,^31^ including, for example, the collection of maternal care expenditure data which may be underestimated; birth rates lower than those reported in other surveys (eg, District Level Household Survey,^27^ National Family Survey)^28^ potentially leading to under-reporting or differential under-reporting of births across income quintiles which may yield overestimation/underestimation of maternal expenditure.^38^ We also assigned an individual income to adolescent girls, whereas they might still depend on their husbands or fathers at this age. In particular, we did not include the potential lifetime economic consequences of adolescent pregnancy such as its impact on school attendance and its long-term impact on earnings losses and poverty,^39^ due to lack of data. Moreover, our analysis only focused on the mortality consequences of adolescent pregnancy and we did not account for the potential sequelae to mothers and children following complicated delivery. Delaying childbirth was modelled as a risk displacement to older women: the elevated risk during adolescence might be a first pregnancy effect or due to unstable relationship and abortion. Such elevated risk is particularly high below age 15 as opposed to that in 15–19 years old; hence, the deaths averted could even be higher if that lower age group was considered in the analysis. Furthermore, we could have examined the variations within the countries, between rural and urban settings, regions and states (eg, India). We used a variety of sources, inputs and surveys from different years. Hence, our analysis aimed at exposing an order of magnitude for the level and distribution of the likely health and financial consequences incurred rather than presenting definitive estimates. Finally, we chose to represent induced poverty in terms of cases of catastrophic health expenditure due to simplicity. Yet issues pertain to its use, notably the choice of an arbitrary threshold (eg, 10%, 20% of income, etc) and the fact that certain individuals (those that forgo healthcare to avoid the likely impoverishing consequences of healthcare spending) might not always be counted in the analysis.^17^

We examined the distribution of maternal-related deaths and impoverishment among pregnant adolescent girls in low-income and middle-income countries with two country cases: Niger and India. Our approach incorporates poverty and distributional analysis in the evaluation of maternal health. This enables a selection of policies based on how much financial risk protection and equity, in addition to how much health, they can provide. Our methodology allows policymakers to consider all of these critical dimensions when making financing decisions. Importantly, it allows them to compare across sectors (eg, education, health) the impact of policies on poverty reduction, which is essential for ministries of finance and development. We show how, in the context of maternal and adolescent health, the equity and poverty alleviation dimensions might be essential and should thus be taken into account, critically pointing to the multifaceted nature of maternal and adolescent health. We found that adolescent pregnancies lead to large equity gaps and impoverishment in low-income and middle-income countries; and that increasing female education could reduce adolescent maternal deaths and provide poverty reduction benefits. In contrast to the distribution of maternal deaths, the OOP costs would accrue more to the richer income groups. This repartition mimicked healthcare usage, which is higher among the rich, as the poor would forgo institutional maternal delivery due to financial constraints, for example. Therefore, well-designed policies across sectors should target the poor and are very important in the context of the Sustainable Development Goals. With universal girl education, large numbers of maternal deaths could be averted, especially among the poor. Large numbers of cases of catastrophic expenditure would be averted: all of them among the poorer in India reinforcing the pro-poor nature of girl education; a majority of them among the richer in Niger pointing to the vast inequalities in access to care. Estimating such catastrophic health expenditure can provide evidence on the individuals who would need to be targeted for maternal services in low-income and middle-income countries. Cash transfers and transport vouchers may be required for the poorest individuals to overcome their financial constraints to access health services and to give poor women the choice to seek care.^38^ ^40^ ^41^

## References

[1] United Nations. Millennium development goals and beyond 2015. http://www.un.org/millenniumgoals/bkgd.shtml (accessed 4 Dec 2015).

[2] United Nations Maternal Mortality Estimation Inter-agency Group. Maternal Mortality Estimates. http://www.maternalmortalitydata.org (accessed 4 Dec 2015).

[3] Alkima L, Chou D, Hogan D Global, regional, and national levels and trends in maternal mortality between 1990 and 2015, with scenario-based projections to 2030: a systematic analysis by the UN Maternal Mortality Estimation Inter-Agency Group. Lancet 2016;387:462–74. 10.1136/bmj.328.7449.1152-a 26584737 PMC5515236

[4] United Nations, Department of Economic and Social Affairs. Population Division, World Population Prospects, the 2015 revision. http://esa.un.org/wpp/ (accessed 4 Dec 2015).

[5] Mayor S Pregnancy and childbirth are leading causes of death in teenage girls in developing countries. BMJ 2004;328:1152 10.1136/bmj.328.7449.1152-a PMC411126 15142897

[6] Institute for Health Metrics and Evaluation. GBD cause patterns. Seattle, WA: Institute for Health Metrics and Evaluation, University of Washington, 2015 http://vizhub.healthdata.org/gbd-compare/ (accessed 4 Dec 2015).

[7] Huang W The impact of fertility changes on maternal mortality. PhD thesis, London School of Hygiene and Tropical Medicine, 2011 http://researchonline.lshtm.ac.uk/682434 (accessed 20 Sep 2014).

[8] Gakidou E, Cowling K, Lozano R Increased educational attainment and its effect on child mortality in 175 countries between 1970 and 2009: a systematic analysis. Lancet 2010;376:959–74. 10.1016/S0140-6736(10)61257-3 20851260

[9] UNICEF. UNICEF data: Monitoring the situation of children and women. http://data.unicef.org/child-protection/child-marriage.html (accessed 11 Dec 2015).

[10] World Bank. World development indicators 2015. Washington DC: World Bank, 2015 http://data.worldbank.org/data-catalog/world-development-indicators (accessed 4 Dec 2015).

[11] Dixon-Mueller R How young is “too young”? Comparative perspectives on adolescent, sexual, marital, and reproductive transitions. Stud Fam Plann 2008; 39:247–62. 19248713 10.1111/j.1728-4465.2008.00173.x

[12] Filippi V, Ronsmans C, Campbell OMR Maternal health in poor countries: the broader context and a call for action. Lancet 2006;368:1535–41. 10.1016/S0140-6736(06)69384-7 17071287

[13] Langer A, Meleis A, Knaul FM Women and health: the key for sustainable development. Lancet 2015;386:1165–210. 10.1016/S0140-6736(15)60497-4 26051370

[14] Lloyd CB, Mensch BS Marriage and childbirth as factors in dropping out from school: an analysis of DHS data from sub-Saharan Africa. Popul Stud (Camb) 2008;62:1–13. 10.1080/00324720701810840 18278669

[15] Arsenault C, Fournier P, Philibert A Emergency obstetric care in Mali: catastrophic spending and its impoverishing effects on households. Bull World Health Organ 2013;91:207–16. 10.2471/BLT.12.108969 23476093 PMC3590618

[16] Powell-Jackson T, Hoque ME Economic consequences of maternal illness in rural Bangladesh. Health Econ 2012;21:796–810. 10.1002/hec.1749 21557382

[17] Wagstaff A Measuring financial protection in health. In: Smith PC, Mossialos E, Papanicolas I, Leatherman S, eds. Performance measurement for health system improvement. Cambridge: Cambridge University Press, 2010:114–37.

[18] World Health Organization. World Health Report 2010—Health Systems Financing, the path to universal coverage. Geneva: World Health Organization, 2010. 10.2471/BLT.10.078741 PMC2878164 20539847

[19] Boerma T, Eozenou P, Evans D Monitoring progress towards universal health coverage at country and global levels. PLoS Med 2014;11:e1001731 10.1371/journal.pmed.1001731 25243899 PMC4171369

[20] Jamison DT, Breman JG, Measham AR Disease control priorities in developing countries. 2nd edn Washington DC: Oxford University Press and the World Bank, 2006. 21250309

[21] Verguet S, Laxminarayan R, Jamison DT Universal public finance of tuberculosis treatment in India: an extended cost-effectiveness analysis. Health Econ 2015;24:318–22. 10.1002/hec.3019 24497185

[22] Verguet S, Gauvreau CL, Mishra S The consequences of tobacco tax on household health and finances in rich and poor smokers in China: an extended cost-effectiveness analysis. Lancet Glob Health 2015;3:e206–16. 10.1016/S2214-109X(15)70095-1 25772692

[23] Verguet S, Olson ZD, Babigumira JB, *et al*. Health gains and financial risk protection afforded by public financing of selected interventions in Ethiopia: an extended cost-effectiveness analysis. *Lancet Global Health* 2015;3:e288–96. 10.1016/S2214-109X(14)70346-8 25889470

[24] Salem ABZ, Mount TD A convenient descriptive model of income distribution: the gamma density. Econometrica 1974;42:1115–27. 23.

[25] Kemp-Benedict E Income distribution and poverty—Methods for using available data in global analysis. May 17, 2001 http://gdrs.sourceforge.net/docs/PoleStar_TechNote_4.pdf (accessed 10 Nov 2015).

[26] Institut National de la Statistique (INS) et ICF International. Enquête Démographique et de Santé et à Indicateurs Multiples du Niger 2012. Calverton, MD: INS et ICF International, 2013.

[27] International Institute for Population Sciences (IIPS). District Level Household and Facility Survey (DLHS-3), 2007–08: India. Mumbai: IIPS, 2010.

[28] International Institute for Population Sciences (IIPS). National Family Health Survey (NFHS-3), 2005–06: India. Mumbai: IIPS, 2007.

[29] Storeng KT, Baggaley RF, Ganaba R Paying the price: the cost and consequences of emergency obstetric care in Burkina Faso. Soc Sci Med 2008;66:545–57. 10.1016/j.socscimed.2007.10.001 18061325

[30] Perkins M, Brazier E, Themmen E Out-of-pocket costs for facility-based maternity care in three African countries. Health Policy Plan 2009;24:289–300. 10.1093/heapol/czp013 19346273 PMC2699243

[31] National Sample Survey Organisation, National Sample Survey Round 60. Ministry of Statistics and Programme Implementation. New Delhi: Government of India, 2004.

[32] Institute for Health Metrics and Evaluation. Educational attainment and child mortality estimates by country 1970–2009. Seattle, USA: Institute for Health Metrics and Evaluation, 2010.

[33] UNESCO. Pricing the right to education: the cost of reaching newt targets by 2030. Education for all global monitoring report policy paper 18. http://unesdoc.unesco.org/images/0023/002321/232197E.pdf (accessed 4 Dec 2015).

[34] Basu AM Why does education lead to lower fertility? A critical review of some of the possibilities. World Dev 2002;30: 1779–90.

[35] Drèze J, Murthi M Fertility, education and development: further evidence from India. 1999 http://www.histecon.magd.cam.ac.uk/docs/female.pdf

[36] Behrman JA Does schooling affect women's desired fertility? Evidence from Malawi, Uganda, and Ethiopia. Demography 2015;52:787–809. 10.1007/s13524-015-0392-3 25951799

[37] Gupta N, Mahy M Adolescent childbearing in sub-Saharan Africa: can increased schooling alone raise ages at first birth? Demogr Res 2003;8:93–106.

[38] Bonu S, Bhushan I, Rani M Incidence and correlates of ‘catastrophic’ maternal health care expenditure in India. Health Policy Plan 2009;24:445–56. 10.1093/heapol/czp032 19687135

[39] Dahl GB Early teen marriage and future poverty. Demography 2010;47:689–718. 20879684 10.1353/dem.0.0120 PMC3000061

[40] Mohanty SK, Srivastava A Out-of-pocket expenditure on institutional delivery in India. Health Policy Plan 2013;28:247–62. 10.1093/heapol/czs057 22709923

[41] Lim SS, Dandona L, Hoisington JA India's Janani Suraksha Yojana, a conditional cash transfer programme to increase births in health facilities: an impact evaluation. Lancet 2010;375: 2009–23. 10.1016/S0140-6736(10)60744-1 20569841

[42] Prual A, Bouvier-Colle MH, de Bernis L Severe maternal morbidity from direct obstetric causes in West Africa: incidence and case fatality rates. Bull World Health Organ 2000;78:593–602. 41. 10859853 PMC2560760

