# Supplementary material for: Maternal-related deaths and impoverishment among adolescent girls in India and Niger: findings from a modelling study
Source: BMJ Open. 2016 Sep 23;6(9):e011586. doi: 10.1136/bmjopen-2016-011586 (PMC5051405; doi:10.1136/bmjopen-2016-011586)
Supplement: Supplementary appendix [file bmjopen-2016-011586supp_appendix.pdf]

Supplementary appendix

Maternal-related deaths and impoverishment among adolescent girls in India and Niger:

findings from a modeling study

by

Stéphane Verguet, Arindam Nandi, Véronique Filippi, Donald Bundy

1. Estimation of adolescent maternal-related deaths, out-of-pocket costs, and impoverishment

This section describes the methods we used for the estimation of adolescent maternal-related deaths, incurred out-of-pocket (OOP) costs, and induced impoverishment. We estimated the level and distribution (across income quintiles and five ages) of:

(1) the number of adolescent maternal-related deaths;

(2) the incurred OOP costs;

(3) the number of cases of catastrophic health expenditure induced.

In a given country C, we divided the adolescent population in five income groups i and five age groups j (15, 16, 17, 18, and 19 year-olds). All the symbols used are listed and defined in Table S1.

1.1. Adolescent maternal-related deaths

Per year, for country C (either India or Niger), we denoted: (i) MMR_C, the maternal mortality ratio (MMR) per 100,000 live births among women aged 15-49 years (174 in

India and 553 in Niger, respectively; Table 1 in the main text); (ii) $Pop_{j,C}$, the number of adolescent women of ages 15, 16, 17, 18 and 19 (58.4 millions divided by 5 in India and 1.0 million divided by 5 in Niger; Table 1 in the main text); (iii) $RR_{j,C}$, the relative risk (compared with 20-24 year-old women) of maternal mortality across the ages 15, 16, 17, 18, and 19 ({4.6,1.0,1.0,1.0,1.0}, Table 1 in the main text); (iv) $AP_{j,C}$, the percent of women aged 15, 16, 17, 18, and 19 pregnant ({1,3,5,9,12}% in India and {3,12,16,19,18}% in Niger; Table 1 in the main text); and (v) $PQ_{i,C}$, the percent of women in income quintile i pregnant ({19,17,13,8,3}% in India and {41,43,37,32,19}% in Niger; Table 1 in the main text).

Subsequently, we estimate the number of adolescent maternal deaths in income quintile i and among age group j in country C as:

$$AD_{i,j,C} = \frac{1}{5} * Pop_{j,C} * MMR_C * RR_{j,C} * AP_{j,C} * \frac{PQ_{i,C}}{Mean(PQ_{i,C})}. \qquad (1)$$

1.2. Adolescent maternal-related out-of-pocket costs

For country C, we denote: (i) $OOP_{DM,C,i}$, the OOP direct medical costs due to complicated deliveries faced by adolescents in income quintile i (\${58,62,70,81,108} in India and \${97,127,140,124,152} in Niger; Table 1 in the main text); (ii) $OOP_{Tr,C,i}$, the OOP transport costs due to complicated deliveries faced by adolescents in income quintile i (about \$8 in India and \$4 in Niger; Table 1 in the main text);[*] (iii) Oc, the occurrence of complicated deliveries (assumed to be 15% across all income quintiles in both countries;

[*]In this analysis, only direct medical costs and transport costs disbursed out of pocket were included.

Table 1 in the main text); and (iv) $u_{C,i}$, healthcare utilization in income quintile i (proxied by skilled birth attendance coverage, {24,34,48,64,85}% in India and {13,19,22,30,71}% in Niger; Table 1 in the main text).

Subsequently, using elements from (1) above, we can estimate the amount of OOP costs incurred by adolescents in income quintile i and age group j as:

$$OOP_{i,j,C} = \frac{1}{5} * Pop_{j,C} * Oc * RR_{j,C} * AP_{j,C} * \frac{PQ_{i,C}}{Mean(PQ_{i,C})} * u_{C,i} * (OOP_{DM,C,i} + OOP_{Tr,C,i}).$$

(2)

1.3. Adolescent maternal-related impoverishment

In country C, adolescent medical impoverishment was quantified by the estimated number of cases of catastrophic health expenditure incurred, which depended on assumed income y and OOP costs (see section 1.2). A case of catastrophic expenditure was counted when OOP costs were found to be higher than 10% of income y. Specifically, we estimated the number of adolescents, per income quintile, for whom the size of OOP costs (sum of direct medical costs and transportation costs) would exceed 10% of their income.

For country C, we derived a distribution of income drawn from a simulated gamma distribution whose shape and scale parameters were based on gross domestic product per capita ($1596 for India and $427 for Niger, respectively; Table 1 in the main text) and Gini coefficient (0.34 for India and 0.32 for Niger; Table 1 in the main text) [1-3]. Subsequently, for each complicated delivery with incurred OOP costs, we assigned an annual income y, based on that derived income distribution. The annual income was also

used to define the income quintile into which each individual belonged (Table 1 in the main text).

Finally, per income quintile, we calculated the number of cases of catastrophic health expenditure occurring. This was done by combining the estimate of annual income with OOP incurred costs estimated (section 1.2); in other words, we counted the number of adolescent women whose OOP costs were larger than $0.10 * y$.

Table S1. Symbols used in the modeling and corresponding definitions.

Symbol	Definition
y	Individual income
Oc	Occurrence of complicated delivery
u_i	Healthcare utilization i.e. skilled birth attendance coverage in income quintile *i*
$OOP_{DM,i}$	Out-of pocket direct medical costs for complicated delivery conducted with skilled birth attendance in income quintile *i*
$OOP_{Tr,i}$	Out-of pocket costs for transport associated with complicated delivery conducted with skilled birth attendance in income quintile *i*
AP_j	Pregnancy rate among adolescents in age group *j* (15, 16, 17, 18, 19 year-olds)
PQ_i	Pregnancy rate among adolescents in income quintile *i*
RR_j	Relative risk of maternal mortality for adolescents (ages *j* = 15, 16, 17, 18, 19) as compared with older age groups (ages 20-24)
MMR	Maternal mortality ratio (per 100,000 live births)
Pop_j	Number of adolescent women among age group *j* (15, 16, 17, 18, 19 year-olds)
$AD_{i,j}$	Adolescent maternal deaths in income quintile *i* and age group *j*
$OOP_{i,j}$	Adolescent maternal out-of-pocket costs in income quintile *i* and age group *j*
$CHE_{i,j}$	Catastrophic health expenditure in income quintile *i* and age group *j*
Eff	Impact of increased education on reducing teenage pregnancy rate

2. Linear relationship between increases in female education levels and adolescent pregnancy rate

We examined the relationship between mean years of education among women aged 15-44 (denoted Edu) [4] and adolescent (15-19 year-olds) pregnancy rate (denoted TP) (percentage of women aged 15-19 who have had children or are currently pregnant), in a given low- and middle-income country [3], controlling for gross domestic product per capita ($GDPc$) and additional variables (Table S2), using the following type of linear model:

$$\ln(TP) = \alpha_0 + \alpha_1 Edu + \alpha_2 \ln(GDPc) + \varepsilon \,. \qquad (3)$$

The complete results of the linear models tried are given in Table S2. For our analysis we retained model (4) where $\alpha_1 = -0.18$. This meant that an increase by 1 year of the mean number of years of education among women aged 15-44 would lead to a relative decrease of the adolescent pregnancy rate of 18% in a given country.

Table S2. Least squares regression of the logarithm of teenage pregnancy rate (percentage of women aged 15-19 who have had children or are currently pregnant) in low- and middle-income countries.

	(1)		(2)		(3)		(4)	
	Coeff.	SE	Coeff.	SE	Coeff.	SE	Coeff.	SE
Mean years of education among 15-44 year-old women	-0.11***	0.02	-0.11***	0.02	-0.15***	0.02	-0.18***	0.02
Logarithm of per capita GDP	0.00	0.05	0.02	0.06	0.02	0.08	0.17*	0.10
Percent of rural population			0.00	0.00	-0.00	0.00	0.00	0.00
Percent of households with female head					0.01	0.00	0.01*	0.01
Percent of women in parliament							-0.01	0.01
Constant	3.43***	0.31	3.26***	0.52	3.55***	0.66	2.57***	0.86
Number of observations	216		216		147		98	
R^2	0.30		0.30		0.42		0.48	

Note: data are from the World Bank database, http://data.worldbank.org/, and the Institute of Health Metrics and Evaluation, http://ghdx.healthdata.org/ihme_data. Years 1970-2009 are included. The following low- and middle-income countries are included: Armenia, Azerbaijan, Burundi, Benin, Burkina Faso, Bangladesh, Bolivia, Botswana, Côte d'Ivoire, Cameroon, Congo, Colombia, Dominican Republic, Ecuador, Egypt, Ethiopia, Georgia, Ghana, Guinea, Guatemala, Honduras, Indonesia, India, Jamaica, Jordan, Kazakhstan, Kyrgyzstan, Cambodia, Laos, Liberia, Sri Lanka, Lesotho, Morocco, Moldova, Madagascar, Mali, Mozambique, Mauritania, Malawi, Namibia, Niger, Nigeria, Nicaragua, Nepal, Pakistan, Peru, Philippines, Paraguay, Romania, Rwanda, Senegal, Sierra Leone, El Salvador, Swaziland, Chad, Togo, Tajikistan, Tunisia, Turkey, Tanzania, Uganda, Ukraine, Vietnam, South Africa, Democratic Republic of the Congo, Zambia, Zimbabwe. Outcome variable is the percentage of women ages 15-19 who have had children or are currently pregnant. Standard errors are heteroskedasticity-robust. Coefficients which are statistically significant at 10%, 5%, and 1% level are marked with *, **, and *** respectively.

GDP = gross domestic product (current US$); Coeff. = regression coefficient; SE = standard error.

3. Estimation of adolescent maternal-related deaths, out-of-pocket costs, and impoverishment averted

We started from the estimations derived in section 1, i.e. from the estimations of the level and distribution (per income quintile and age) of: the number of adolescent maternal deaths (section 1.1 and equation 1); the OOP costs incurred (section 1.2 and equation 2); and the number of cases of catastrophic health expenditure induced (section 1.3). Subsequently, we applied the impact estimated in section 2 (e.g. $\alpha_1 = -0.18$ or 18% reduction) to the estimates of the base case scenario (section 1). This yielded: adolescent maternal-related deaths averted, adolescent OOP costs averted, and adolescent catastrophic health expenditure averted, amounting to 18% of what was estimated in the base case scenario (section 1).

4. Sensitivity analysis

We conducted a Monte Carlo probabilistic sensitivity analysis to estimate aggregate uncertainty from key inputs. Parameters were given values using probability distributions (details are given in Table S3).

We also pursued univariate sensitivity analyses where: (1) different thresholds (20% and 40%) for the catastrophic health expenditure were used (Tables S4 and S5); (2) a poverty headcount (individuals crossing the country poverty line due to OOP costs) in lieu of catastrophic health expenditure was used (Tables S4 and S5); (3) a different size (smaller 11% relative reduction instead of 18%, as given in Table S2) for the impact of increased female education on the adolescent pregnancy rate was used (Tables S6 and S7); and (4) benefits and costs were discounted at 3% per year over five subsequent years (corresponding to the ages 15, 16, 17, 18, and 19) (Tables S8 and S9).

Table S3. Probability distributions of the key inputs for the analysis.

Input	Parameters
Out-of-pocket cost for complicated delivery ($)	Gamma with: Mean = {97; 127; 140; 124; 152} and SD = {3; 4; 4; 4; 4} for Niger Mean = {58; 62; 70; 81; 108} and SD = {2; 2; 2; 2; 3} for India
Out-of-pocket cost for transport ($)	Gamma with: Mean = 4 and SD = 0.1 for Niger Mean = {8; 8; 8; 8; 6} and SD = {0.2; 0.2; 0.2; 0.2; 0.2} for India
Maternal mortality ratio (per 100,000 live births)	Beta with Mean = 553 and SD = 102 for Niger Beta with Mean = 174 and SD = 22 for India
Relative risk of maternal mortality per age (15, 16, 17, 18, 19)	Logistic with Mean = {4.6, 1.0, 1.0, 1.0, 1.0} SD = {0.66, 0.07, 0.07, 0.07, 0.07}
Effect of 1-year increase in female education on teenage pregnancy rate	Beta with Mean = 0.18, SD = 0.02

SD, standard deviation.

Table S4. Adolescent impoverishment induced in Niger, using a variety of metrics: cases of catastrophic health expenditure with a threshold of 10% (base case); cases of catastrophic health expenditure with a threshold of 20%; cases of catastrophic health expenditure with a threshold of 40%; and poverty cases using the national poverty line ($316 annual).

Outcome	Total	Income quintile I	Income quintile II	Income quintile III	Income quintile IV	Income quintile V
Catastrophic health expenditure, 10% threshold	6150 (5370-6900)	730 (640-830)	1140 (1000-1280)	1110 (980-1250)	1320 (1160-1490)	1840 (1550-2060)
Catastrophic health expenditure, 20% threshold	5320 (3610-6210)	730 (650-820)	1130 (1000-1270)	1120 (980-1260)	1340 (70-1490)	1000 (0-1650)
Catastrophic health expenditure, 40% threshold	2250 (1100-3060)	740 (1200-1900)	1120 (500-2800)	390 (0-2600)	0	0
Poverty cases	1170 (800-1560)	0	0	620 (540-700)	560 (210-910)	0

95% uncertainty ranges in parentheses.

Table S5. Adolescent impoverishment induced in India, using a variety of metrics: cases of catastrophic health expenditure with a threshold of 10% (base case); cases of catastrophic health expenditure with a threshold of 20%; cases of catastrophic health expenditure with a threshold of 40%; and poverty cases using the national poverty line ($787 annual).

Outcome	Total	Income quintile I	Income quintile II	Income quintile III	Income quintile IV	Income quintile V
Catastrophic health expenditure, 10% threshold	28,620 (16,180-52,590)	28,620 (16,180-52,590)	0	0	0	0
Catastrophic health expenditure, 20% threshold	7100 (3520-13,440)	7100 (3520-13,440)	0	0	0	0
Catastrophic health expenditure, 40% threshold	1510 (710-2780)	1510 (710-2780)	0	0	0	0
Poverty cases	11,560 (8970-14,250)	0	11,560 (8970-14,250)	0	0	0

95% uncertainty ranges in parentheses.

Table S6. Impact of increasing mean years of female education by 1 year in Niger (with a smaller impact of 11% relative reduction in lieu of 18%): number of adolescent maternal deaths averted, amount of adolescent out-of-pocket costs averted, and number of adolescent catastrophic health expenditure averted, per income quintile.

Outcome	Total	Income quintile I	Income quintile II	Income quintile III	Income quintile IV	Income quintile V
Maternal deaths averted	100 (50-150)	25 (10-35)	25 (10-40)	20 (10-30)	20 (10-30)	10 (5-15)
Out-of-pocket costs averted (1000s of 2014 US$)	93 (58-136)	8 (5-13)	16 (9-25)	18 (10-28)	19 (11-30)	32 (18-50)
Cases of catastrophic health expenditure averted	680 (450-940)	80 (50-110)	120 (80-170)	120 (80-170)	150 (100-200)	200 (130-280)

95% uncertainty ranges in parentheses.

Table S7. Impact of increasing mean years of female education by 1 year in India (with a smaller impact of 11% relative reduction in lieu of 18%): number of adolescent maternal deaths averted, amount of adolescent out-of-pocket costs averted, and number of adolescent catastrophic health expenditure averted, per income quintile.

Outcome	Total	Income quintile I	Income quintile II	Income quintile III	Income quintile IV	Income quintile V
Maternal deaths averted	760 (460-1150)	240 (150-360)	220 (130-330)	160 (100-240)	100 (60-160)	40 (25-60)
Out-of-pocket costs averted (1000s of 2014 US$)	1860 (1200-2710)	260 (150-400)	370 (230-570)	450 (260-690)	460 (260-700)	330 (190-510)
Cases of catastrophic health expenditure averted	3190 (1530-6040)	3190 (1530-6040)	0	0	0	0

95% uncertainty ranges in parentheses.

Table S8. Discounted benefits and costs of increasing mean years of female education by 1 year in Niger: number of adolescent maternal deaths averted (discounted), amount of adolescent out-of-pocket costs averted (discounted), and number of adolescent catastrophic health expenditure averted (discounted), per income quintile.

Outcome	Total	Income quintile I	Income quintile II	Income quintile III	Income quintile IV	Income quintile V
Maternal deaths averted	149	37	37	32	28	16
Out-of-pocket costs averted (1000s of 2014 US$)	133	11	24	26	27	46
Cases of catastrophic health expenditure averted	976	116	179	177	211	292

The discount rate used was 3% per year. Benefits and costs incurred were thus multiplied by 1 in year 1, by 0.97 in year 2, 0.94 in year 3, 0.92 in year 3 and 0.89 in year 4.

Table S9. Discounted benefits and costs of increasing mean years of female education by 1 year in India: number of adolescent maternal deaths averted (discounted), amount of adolescent out-of-pocket costs averted (discounted), and number of adolescent catastrophic health expenditure averted (discounted), per income quintile.

Outcome	Total	Income quintile I	Income quintile II	Income quintile III	Income quintile IV	Income quintile V
Maternal deaths averted	1156	366	330	240	157	63
Out-of-pocket costs averted (1000s of 2014 US$)	2834	396	567	681	692	498
Cases of catastrophic health expenditure averted	4785	4785	0	0	0	0

The discount rate used was 3% per year. Benefits and costs incurred were thus multiplied by 1 in year 1, by 0.97 in year 2, 0.94 in year 3, 0.92 in year 3 and 0.89 in year 4.

References

1. Salem ABZ, Mount TD. A Convenient Descriptive Model of Income Distribution: The Gamma Density, Econometrica 1974; 42(6):1115-1127.
2. Kemp-Benedict E. Income distribution and poverty – Methods for using available data in global analysis. May 17, 2001. Available from: http://gdrs.sourceforge.net/docs/PoleStar_TechNote_4.pdf (accessed November 10, 2015).
3. World Bank. World Development Indicators 2015. Washington, DC: World Bank, 2015.
4. Institute for Health Metrics and Evaluation. Educational Attainment and Child Mortality Estimates by Country 1970-2009. Seattle, United States: Institute for Health Metrics and Evaluation, 2010.
